# The Microevolution of Antifungal Drug Resistance in Pathogenic Fungi

**DOI:** 10.3390/microorganisms11112757

**Published:** 2023-11-13

**Authors:** Kylie J. Boyce

**Affiliations:** School of Science, RMIT University, Melbourne, VIC 3085, Australia; kylie.boyce@rmit.edu.au; Tel.: +61-(0)3-9925-7101

**Keywords:** fungi, pathogen, antifungal drugs, resistance, microevolution, adaptation, mutator, ploidy, aneuploidy, phenotypic diversity

## Abstract

The mortality rates of invasive fungal infections remain high because of the limited number of antifungal drugs available and antifungal drug resistance, which can rapidly evolve during treatment. Mutations in key resistance genes such as *ERG11* were postulated to be the predominant cause of antifungal drug resistance in the clinic. However, recent advances in whole genome sequencing have revealed that there are multiple mechanisms leading to the microevolution of resistance. In many fungal species, resistance can emerge through *ERG11*-independent mechanisms and through the accumulation of mutations in many genes to generate a polygenic resistance phenotype. In addition, genome sequencing has revealed that full or partial aneuploidy commonly occurs in clinical or microevolved in vitro isolates to confer antifungal resistance. This review will provide an overview of the mutations known to be selected during the adaptive microevolution of antifungal drug resistance and focus on how recent advances in genome sequencing technology have enhanced our understanding of this process.

## 1. Introduction

Fungal infections pose an escalating health problem; however, their contribution to the global burden of disease remains under-recognized. It has been estimated that 1.7 billion people are infected with fungi each year, resulting in 1.5 million deaths annually from invasive infections [1,2]. Although there are more than 600 species of fungi that can cause disease in humans, more than 90% of all reported deaths result from invasive infections with the opportunistic pathogens *Cryptococcus neoformans*, *Candida albicans*, *Aspergillus fumigatus* and *Pneumocystis jirovecii* [1,3]. Other species within the *Cryptococcus* species complex (*C. deneoformans* and *C. gattii*), *Candida* genera (*C. glabrata*, *C. tropicalis*, *C. parapsilosis*, *C. krusei* and *C. auris*) and *Aspergillus* genera (*A. flavus*, *A. terreus*, *A. niger* and *A. nidulans*) also cause human invasive infections [1,2,4,5,6]. In addition, moulds in the *Fusarium*, *Scedosporium*, *Mucorales* and *Lomentospora* genera can cause life-threatening invasive infections, which, although rarer, have high mortality, because of high resistance rates or inherent resistance [7]. Other significant invasive fungal infections are restricted to endemic regions and are caused by infections with thermally dimorphic pathogenic species including *Coccidioides immitis*, *Coccidioides posadasii*, *Blastomyces dermatitidis* and *Histoplasma capsulatum* (USA); *Paracoccidioides brasiliensis* and *Paracoccidioides lutzii* (Brazil); and *Talaromyces marneffei* (Southeast Asia) [8,9,10]. The World Health Organisation (WHO) has identified *C. neoformans*, *C. albicans*, *A. fumigatus* and *C. auris* as a critical group of species requiring priority research development and public health action to improve responses and prevent the development of antifungal drug resistance [7]. *C. glabrata*, *Histoplasma* spp., *Mucorales*, *Fusarium* spp., *C. tropicalis* and *C. parapsilosis* are classified as high priority by the WHO, and *Scedosporium* spp., *Lomentospora prolificans*, *Coccidioides* spp., *C. krusei*, *C. gattii*, *T. marneffei*, *P. jirovecii* and *Paracoccidioides* spp. are classified as medium priority [7].

Invasive fungal infections are difficult to treat and result in a high mortality rate, often surpassing 50% and increasing to up to 90% for some species if treatment is delayed [1,7,11]. The major contributing factors to mortality are the limited number of antifungal drugs available and antifungal drug resistance, which results in ineffectual treatment or relapse [12,13,14,15,16]. Resistance has been described for every class of antifungal drugs, is common in some drug classes such as azoles and evolves rapidly during treatment [13].

**Figure 1:**
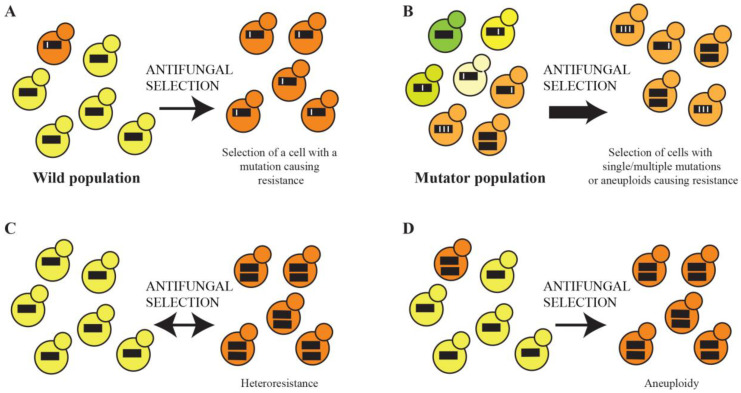
Adaptive evolution of antifungal resistance. (**A**). Cells in the population that possess a mutation resulting in antifungal drug resistance are selected and become predominant in the population. (**B**). An elevated mutation rate provides higher genetic diversity within a population on which selection for antifungal-resistant cells can occur. (**C**). Transient aneuploidy (heteroresistance) occurs in the presence of an antifungal to confer resistance. (**D**). Permanent aneuploidy-conferring antifungal resistance is selected for in a clinical population. The thick black lines represent schematic chromosomes, with white lines representing mutations.

Fungal cells possessing mutations causing antifungal resistance are selected for in the clinic and become predominant in the population in a short timeframe in a process termed adaptive microevolution (Figure 1A). Adaptive microevolution is enhanced by an increased mutation rate, which provides higher genetic diversity within a population on which selection can act (Figure 1B). Isolates with elevated mutation rates, termed mutators, are associated with the enhanced evolution of antifungal resistance [17]. Recent advances in next-generation sequencing technology have revealed that resistance can emerge through single mutations in key genes or via the accumulation of mutations in many genes (polygenic), changes to the transcriptome and aneuploidy (chromosome duplications). In the presence of an antifungal, fungi can undergo a process termed heteroresistance, where transient aneuploidy occurs to confer resistance (Figure 1C). If the aneuploidy becomes permanent, these stable aneuploids are selected for in a clinical population (Figure 1D). This review will provide an overview of the mutations known to be selected during the adaptive microevolution of antifungal drug resistance and focus on how recent advances in technology have enhanced our understanding of this process.

## 2. Antifungal Drugs

**Figure 2:**
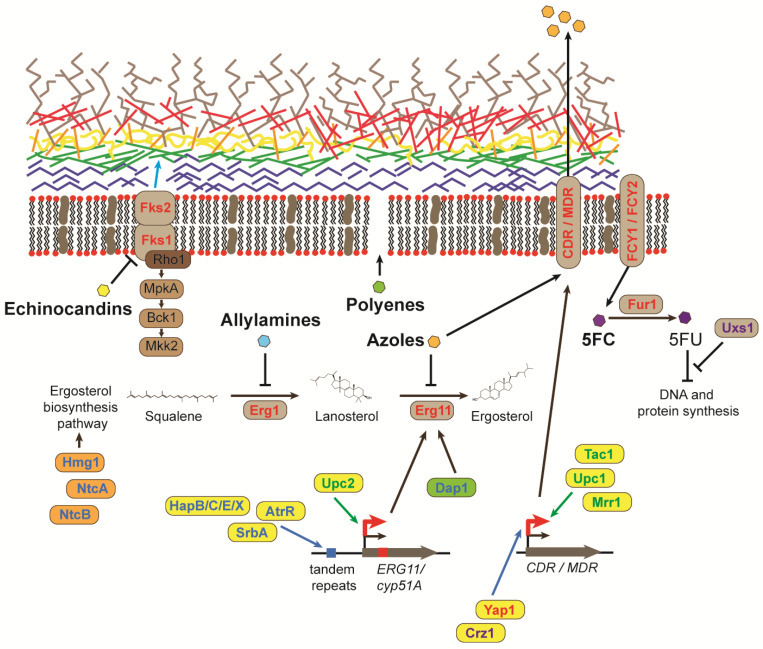
Molecular mechanisms of resistance to antifungal drugs. Schematic of the fungal cell membrane and wall showing the mechanism of action of the five antifungals and the mechanisms of resistance (5FC: 5-fluorocytosine and 5FU: 5-fluorouracil). Mechanisms common to several different fungal species are indicated in red (text, arrows or boxes), those specific to *A. fumigatus* in blue (text and boxes), to *C. neoformans* in purple text and to *Candida* species in green text. The fungal cell wall comprises chitin (blue), ß-1,3-glucan (light green), ß1,6- glucan (orange), proteins (yellow), α-1,3-glucan (red) and galactomannans (brown). Transcription factors and proteins that regulate ergosterol biosynthesis are shown in yellow and orange, respectively. Damage Resistance Protein 1 (Dap1) complex is shown in green.

The limited numbers of antifungal drugs and the tapered pipeline for the development of novel antifungals are widely recognised challenges for clinical mycology [1]. Many antifungal drugs can cause anaphylactic reactions or other life-threatening side effects, including renal or liver damage, and there has only been a single new class of antifungal, the echinocandins, released in the last few decades [18,19]. In addition, the use of antifungal drugs is limited by the type of administration, unfavourable drug interactions, bioavailability in target tissues and restricted activity [1,19]. There are four main classes of antifungals used to treat invasive fungal infections: azoles, polyenes, pyrimidine analogues and echinocandins. A fifth class of antifungals, allylamines, is only used for treating superficial infections [19] (Figure 2).

Azoles are the largest and most widely used class of antifungal agents because of their broad-spectrum activity and oral administration, which is useful in resource-limited settings [1,19]. Azole antifungals inhibit the biosynthesis of ergosterol, a crucial component of the cell membrane, which disrupts fungal growth and replication. Azoles bind the iron in the active site of the enzyme lanosterol 14 alpha-demethylase, causing a block in the ergosterol biosynthesis pathway and the accumulation of toxic sterols [19]. Similar to azoles, allylamine antifungals target ergosterol biosynthesis by inhibiting an essential enzyme, in this case, squalene epoxidase, which leads to the accumulation of squalene and increasing membrane permeability [19,20]. Polyenes are broad-spectrum antifungals that bind ergosterol in the cell membrane, inducing an extramembranous sterol sponge that destabilizes the membrane and generates membrane pores, which causes the leakage of cellular content and death [19,21,22]. However, use is limited by the need for intravenous administration and severe side effects [19]. 5-fluorocytosine (5-FC) is a pyrimidine analogue used in synergistic combination with polyene amphotericin B. 5-FC enters the fungal cell via the cytosine permease enzyme, where it is converted by cytosine deaminase into the active form, 5-fluorouracil (5-FU). 5-FU can compete with uracil to disrupt RNA and subsequent protein synthesis and can also inhibit DNA synthesis through the inhibition of thymidylate synthase. The newest class of broad-spectrum antifungals, echinocandins, inhibit the synthesis of ß-1,3-glucan in the fungal cell wall, which results in osmotic instability and cell death [23]. Use is limited because of poor absorption, a short half-life and the requirement for daily intravenous administration [19].

## 3. Mutations in Genes Involved in Ergosterol Biosynthesis Confer Antifungal Resistance

Resistance to azole antifungals is commonly attributed to the selection of mutants that over-express or alter the *ERG11***/***cyp51A* gene encoding the target enzyme of the ergosterol biosynthesis pathway, lanosterol 14 alpha-demethylase [24,25,26,27,28,29] (Figure 2). Single-nucleotide mutations in *ERG11* have been shown to lead to resistance in *C*. *albicans*, *C. glabrata*, *C. tropicalis*, *C. krusei*, *C. parapsilosis* and *C. albicans* clinical isolates, which typically over-express *ERG11* [24,27,28,30]. *C. auris* clinical isolates possess *ERG11* sequence variants that account for their intrinsic multi-drug resistance to azoles [28,31]. *ERG11* mutations have also been associated with fluconazole resistance in *C. neoformans* clinical isolates [32,33,34,35]. Mutations in other genes of the ergosterol pathway can also lead to azole resistance but are less commonly observed. In *C. glabrata*, the resistance of microevolved strains can occur via mutations in *ERG3* and *ERG4* [30]. In *C. albicans*, mutations in *ERG3*, *ERG2* or *ERG6* confer resistance by preventing the generation of toxic sterol intermediates; however, *ERG3A* and *ERG3B* mutants in *A. fumigatus* do not [36,37].

In aspergillosis patients where an azole-resistant isolate is obtained through environmental exposure, the most common mechanism resulting in resistance is the duplication of a 34 bp tandem repeat in the *cyp51A* (*ERG11* homologue) promoter, in combination with a specific substitution (TR_34_/L98H) [14,38,39]. This mutation was found to be correlated with exposure to agricultural azoles and confers pan-azole resistance [39,40,41]. Agricultural use of the triazoles tebuconazole and propiconazole to control fungal diseases in plant crops has increased and results in the persistent contamination of soil, sewage and wastewater in the environment [40,41,42]. A recent surveillance study in Vietnam, where the use of fungicides is widespread and poorly regulated, showed that azole resistance occurs predominantly in isolates from cultivated soils, and 95.2% of *A. fumigatus* environmental isolates were resistant to at least one azole [42].

More pan-azole resistance alleles arising from various alterations to the *cyp51A* promoter tandem repeat have since been discovered in both the clinic and the environment in various locations around the world [39,40,43]. A recent study analysing 1190 azole-resistant *A. fumigatus* isolates, obtained from the environment from regions all over the globe, predominately carried the *cyp51A* TR_34_/L98H (60.7%) or TR_46_/Y121F/T289A (15.0%) alleles [39]. *cyp51A* mutations that occur during human infection are commonly missense mutations that prevent the azole from binding to the 14 α-demethylase enzyme azole target [6,25,40,44].

Genes regulating the transcription of ergosterol biosynthesis genes and ergosterol production also play a role in azole susceptibility (Figure 2). The sterol regulatory element binding protein (SREBP) pathway is required for adaptation to hypoxia and sterol homeostasis in fungi [45]. Under low oxygen, the SREBP transcription factor in *C. neoformans* (Sre1) and *A. fumigatus* (SrbA) activates genes required for ergosterol biosynthesis, and deletion results in increased susceptibility to azole antifungals [46,47,48]. SrbA has been shown to bind directly to the tandem repeats in the *cyp51A* promoter [6,48]. An additional transcription factor, encoded by *atrR*, also binds the tandem repeats in the *cyp51A* promoter and is required for normal tolerance to azoles [49]. The homologous gene to *SRE1/srbA* in *C. albicans*, *CPH2*, is not necessary for ergosterol biosynthesis [50]. Rather, in *C. albicans*, a different transcription factor, Upc2, regulates the expression of ergosterol biosynthesis genes [51]. The disruption of *UPC2* in *C. albicans* increases azole susceptibility and overexpression or activating mutations cause azole resistance in vitro [51,52]. In *C. albicans* clinical isolates, gain-of-function *UPC2* mutants contribute to the increased expression of *ERG11* and fluconazole resistance [53,54,55,56]. The deletion of *UPC2* in *C. glabrata* decreases the expression of ergosterol biosynthesis genes [57].

*cyp51A* expression in *A. fumigatus* is also regulated by a transcriptional complex containing HapB, HapC and HapE and an additional factor, HapX [38]. A mutation in *hapE* was initially found in an azole-resistant clinical isolate via whole genome sequencing [44]. The deletion of *HapB*, *HapC*, *HapE* and *HapX* and the expression of HapE^P88L^ result in increased resistance to azoles [38]. HMG CoA reductase encoded by *hmg1* is a sterol-sensing protein bound to the endoplasmic reticulum that initiates ergosterol biosynthesis. *hmg1* mutations generated in the sterol-sensing domain or identified in clinical isolates result in altered sterol levels and azole resistance [58,59]. Mutations in the NtcA and NtcB subunits of the Negative co-factor two (Ntc2) complex, which regulates ergosterol biosynthesis, also result in pan-azole resistance [60]. Mutations in subunits of the Damage Resistance Protein 1 (Dap1) complex, which regulates cyp51a and Erg5 function, also result in resistance [61].

Similar to azoles, allylamine antifungals target ergosterol biosynthesis [20] (Figure 2). Terbinafine is an allylamine antifungal commonly used to treat dermatophyte infections. Mutations in the gene encoding the target enzyme (*ERG1*) confer resistance to terbinafine in clinical isolates of *Trichophyton interdigitale* and *Trichophyton rubrum* [62,63]. The introduction of the equivalent mutation in *A. fumigatus* and *C. albicans* also confers terbinafine resistance [62,64].

## 4. The Overexpression of Genes Encoding Efflux Pumps Confers Azole Resistance

The overexpression of *MDR1* genes encoding efflux pumps of the major facilitator superfamily (MFS) or *CDR* genes encoding efflux pumps of the ATP-binding cassette (ABC) superfamily occurs in azole-resistant clinical isolates of *C. albicans*, *C. parapsilosis*, *C. krusei*, *C. auris* and *C. glabrata* [16,65,66] (Figure 2). Long-term therapy of oropharyngeal candidiasis in AIDS patients results in the constitutive expression of the *CDR1*, *CDR2* and *MDR1* genes [67,68]. The transcription factors that control the expression of efflux pumps in *C. albicans* and *C. parapsilosis* are Tac1, Mrr1 and Upc1 [69,70,71]. Another transcription factor, Cap1, cooperates with Mrr1 in *C. albicans* [69]. Cph1 and Mcm1 are additional negative and positive regulators of *MDR1* expression, respectively [72,73]. *C. albicans* and *C. parapsilosis* gain-of-function mutations in *TAC1* result in the constitutive expression of *CDR1* and *CDR2* in azole-resistant clinical isolates and in vitro [29,69,70,71,74]. Likewise, *MRR1*, *UPC1* and *CAP1* gain-of-function mutations result in the overexpression of *MDR1* in azole-resistant clinical isolates (*MRR1* and *UPC1*) or in vitro (*CAP1*) [29,53,69,70,71,75]. Mutations in *TAC1* and *UPC1* and the overexpression of *CDR1* are also responsible for azole resistance in *C. auris* [76,77].

Almost all azole-resistant *C. glabrata* clinical isolates and those from in vitro evolution experiments possess activating mutations in the *PDR1* gene, which encodes a transcription factor that induces the expression of *CDR1* [27,30,78,79,80,81]. Pdr1 is regulated in part by the Hst1 deacetylase, which regulates gene expression by interacting with the mediator complex [81]. The deletion of *HST1* and components of the mediator complex result in fluconazole resistance [82,83].

In *C. neoformans* and *C. gattii*, the expression of the ABC and MFS transporter genes is induced upon treatment with fluconazole [84,85]. *S. cerevisiae*-expressing *C. gattii AFR1*, *AFR2* and *MDR1* lead to higher resistance to fluconazole [84]. *AFR1* is overexpressed in a fluconazole-resistant *C. neoformans* clinical isolate, and mice infected with an *AFR1* mutant respond better to treatment with fluconazole [86,87]. *AFR1* overexpression in a susceptible strain in vitro or gene deletion results in increased fluconazole resistance or susceptibility, respectively [85,87]. The expression of *AFR1* is regulated by the CRZ1 and yap1 transcription factors [85].

The overexpression of efflux pumps encoded by *atrI*, *cdr1B* and *mdr1* in *A. fumigatus*, occurs in azole-resistant clinical isolates and the overexpression of *atrF*, *Afumdr1*, *Afumdr3* and *Afumdr4* in vitro results in azole resistance [6]. Mutations in genes encoding transcription factors *atrR* and *yap1*, which regulate the expression of *cdr1B* and *atrF*, respectively, also confer resistance [88,89].

## 5. Mutations in Genes Encoding Glucan Synthases Result in Resistance to Echinocandin Antifungals

Resistance to echinocandins arises from mutations in the genes encoding the catalytic subunits of the target enzyme, 1,3-ß-D-glucan synthase complex, encoded by *FKS* genes (Figure 2). In *C. albicans*, *C. krusei*, *C. auris* and *C. tropicalis*, mutations specifically in *FKS1* cause resistance [14,28,29,31,90,91]. Single-residue substitutions are commonly located in two *FKS1* hot spots in *C. albicans* at amino acids 641–649 and 1357–1364 [92]. However, additional resistance mechanisms, yet to be identified, must also exist, as most echinocandin-resistant *Candida* isolates lack mutations within *FKS1* [93]. In *C. glabrata*, resistance can be conferred by mutations in either *FKS1* or *FKS2* [14,30]. *C. parapsilosis* and *Candida guilliermondii* are intrinsically resistant to echinocandins because of a single nucleotide polymorphism that occurs in the *FKS1* hotspot that confers resistance in other *Candida* species [94].

Echinocandin resistance in *A. fumigatus* clinical isolates is rare. One mutation in *FKS1* has been found in an *A. fumigatus* echinocandin-resistant clinical isolate after micafungin treatment failure [95].

## 6. Resistance to Polyenes and the Pyrimidine Analogue 5-FC

Amphotericin B resistance is associated with reduced fitness, so, as a consequence, clinical resistance is rare despite over 50 years of use as a monotherapy to treat invasive infections [96,97]. Similar to azoles, resistance can arise through mutations in the *ERG* genes of the ergosterol biosynthesis pathway. Missense mutations in *ERG3* and *ERG6* can confer amphotericin B resistance in *C. glabrata* and *C. auris* [98,99,100]. However, most *Candida* amphotericin B-resistant strains have not been characterised at the gene level but rather by detecting changes in the sterol composition of membranes. The only amphotericin B-resistant *C. neoformans* isolate carries a mutation in *ERG2* [101].

5-FC is used only in combination with amphotericin B, as resistance to 5-FC emerges frequently. Mutations in *FCY1* and *FCY2*, permeases required for 5-FC transport, and in *FUR1*, a gene that encodes a uracil phosphoribosyltransferase that converts 5-FC into toxic 5-FU, confer resistance to 5-FC in *C. albicans*, *C. glabrata*, *C. auris* and *C. neoformans* [31,102]. Mutations in a gene encoding an enzyme that converts UDP-glucuronic acid into UDP-xylose (*UXS1*), which results in altered nucleotide metabolism, also confers resistance in *C. neoformans* by suppressing the toxicity of 5-FC and its derivative, 5-FU [103] (Figure 2).

## 7. Mutation Rate Enhances the Microevolution of Drug Resistance

The microevolution of antifungal resistance is significantly increased by an elevated mutation rate, which bestows the fungal population with higher genetic diversity upon which selection can act [17]. Strains that exhibit an elevated mutation rate, termed mutators, exhibit the rapid emergence of azole resistance in vitro in *C. neoformans*, *Cryptococcus deuterogattii*, *C. glabrata* and *A. fumigatus* [104,105,106,107]. In clinical populations, the most frequently mutated gene giving rise to a mutator phenotype is the *MSH2* gene of the DNA mismatch repair (MMR) pathway, although an *MLH1* variant (also in the MMR pathway) has been found in *C. auris* [108]. Non-synonymous variation in *MSH2* has been discovered in clinical populations of *C. deuterogattii*, *C. neoformans*, *C. glabrata* and *A. fumigatus* [12,104,105,106,107,109,110,111,112,113,114]. However, the exact prevalence of *MSH2* mutators in clinical populations and their clinical relevance remains controversial, as a correlation with antifungal resistance is often not found. Challenges in measuring mutation rates have led to many studies reporting sequence variance without confirming an increased mutation rate, and some *MSH2* alleles in *C. glabrata* previously called mutators have subsequently been shown not to result in a mutator phenotype [105,111,112,113,114,115]. Nevertheless, mutations in *MSH2* are strongly associated with the emergence of resistance to azoles, polyenes, pyrimidine analogues and echinocandins in vitro [104,105,106,107]. In *C. glabrata*, resistance to azoles, amphotericin B and echinocandins in *msh2*Δ mutants can arise from mutations in *PDR1* (azoles), *ERG6* (amphotericin B), *FKS1* and *FKS2* (echinocandins) [105]. Whole genome sequencing of 5-FC-resistant *msh2*Δ mutants in *C. deuterogattii* revealed mutations in *FUR1*, *FCY2* and *UXS1* [103]. Whole genome sequencing of azole and amphotericin B-resistant *msh2*Δ mutants in *C. neoformans* revealed polygenic resistance, where mutations accumulate in genes that alter stress signalling, cellular efflux, membrane trafficking and epigenetic modification [116].

## 8. Whole Genome Sequencing Reveals That the Microevolution of Drug Resistance Can Be Polygenic

Although mutations in single key genes such as *ERG11* and *PDR1* appear to be the predominant mode of azole resistance in *C. albicans* and *C. glabrata*, respectively, this is likely not the case in other pathogenic fungi, where resistance is also driven by *ERG11*-independent mechanisms [27,117]. Between 50 and 70% of fluconazole-resistant *C. neoformans* clinical isolates lack any mutations in *ERG11* [26,32,33,34,35]. A recent study of the *C. gattii* Pacific Northwest outbreak also concluded that neither the overexpression of *ERG11* nor mutations within the gene were responsible for the resistance to fluconazole in these isolates [118]. In addition, greater than 50% of *A. fumigatus* azole-resistant clinical isolates do not possess mutations in the regulatory or coding regions of *cyp51A* [6,39,65,119,120]. Recent advances in next-generation sequencing technology have enabled more studies utilizing mutational profiling to follow the emergence of antifungal drug resistance. Genome sequencing of clinical isolates over the course of infection has been performed, as well as of resistant isolates generated from in vitro microevolution experiments, where isolates are passaged in a laboratory in low concentrations of antifungals. These types of studies have revealed that resistance likely emerges through the accumulation of mutations in many genes (e.g., a polygenic phenotype).

An analysis of the mutational profiles of *C. albicans* clinical isolates from oral candidiasis patients revealed mutations in genes required for filamentous growth, cell adhesion, biofilm formation, cell cycle and stress, drug responses and carbohydrate binding, as well as changes in ploidy [121]. Transcriptomic analysis of the evolution of a *C. glabrata* clinical isolate over time from azole susceptibility to posaconazole resistance and clotrimazole resistance to fluconazole/voriconazole resistance showed that only the population with resistance to all azoles had a gain-of-function *PDR1* mutation, whereas intermediate strains possessed alternative resistance mechanisms [122]. In *C. auris*, the mutational spectrum, coupled with an analysis of the transcriptome of fluconazole-resistant in vitro microevolved strains, suggests mutations commonly accumulate in genes encoding transcription factors (*TAC1B*, *UPC2*, *ZCF18* and *ZCF22*), but there are a large number of different mechanisms that promote drug resistance, including changes in ploidy and multiple pathways leading to resistance, including efflux transporter upregulation and transcriptional changes in ribosome biogenesis, RNA metabolism and sugar transport [77,108,123].

Whole genome sequencing of in vitro microevolved azole-resistant *msh2* (mutator) isolates in *C. neoformans* has shown aneuploidy and mutations accumulating in the genes of some of the same biological processes shown by transcriptomic studies to be differentially expressed in response to azole exposure, such as stress signalling, transmembrane transport, epigenetic modification, translation, transcription and carbohydrate metabolism [116,124]. Azole-resistant microevolved strains accumulated mutations in genes that encode the components required for membrane trafficking (*KES1* and *ALP3*) and epigenetic modification (*RLF2*, *EAF1*, *EAF6*, *YAF9* and *SWC4*), and the deletion of these genes resulted in fluconazole resistance [116].

In vitro microevolution experiments on voriconazole resistance in *A. fumigatus* showed resistant strains did not possess mutations in *cyp51A*, *hmg1* or *hapE*, but transcriptomic analysis of these strains showed resistance was likely due to the overexpression of transcription factor *asg1*, which has been shown to regulate the expression of several ABC and MFS transporter genes and genes of the ergosterol biosynthesis pathway [125].

## 9. Heteroresistance Caused by Transient Aneuploidy and Permanent Aneuploidies in Clinical Isolates

Recent advances in whole genome sequencing and mutational profiling have also revealed that large-scale alterations to the genome, such as changes in ploidy (the number of chromosome sets), are a common mechanism utilized by fungi to adapt to environmental stress and generate azole resistance [121,126,127,128]. Exposure to azole antifungals has been shown to result in transient aneuploidy in *C. neoformans* in a process called heteroresistance [129,130]. Upon exposure to azoles, one or more aneuploidies (chromosome duplications) rapidly develop; however, normal ploidy is re-established when the azole is removed because of reduced fitness [127,129,130]. In response to increasing fluconazole concentrations in *C. neoformans*, chromosome 1 containing *ERG11* and *AFR1* is duplicated, followed by the subsequent duplication of chromosomes 4, 10 and 14 [127]. The transient aneuploidy of chromosome 1 is concomitant with increased fluconazole MIC and clinical relapse in cryptococcal meningitis patients [131].

In addition, whole genome sequencing has revealed many antifungal-resistant clinical isolates possess permanent aneuploidies. *C. neoformans* clinical isolates exposed to azoles commonly have disomy of chromosome 1, which results in azole resistance [127,131,132,133]. Exposure to fluconazole in vitro rapidly leads to entire or segmental disomy of chromosome 1 (92% of isolates) and chromosome 4 (36%) in combination with other disomies [134]. Aneuploidy of other chromosomes (2, 4, 6, 8–10, 12–14) has also been observed [12,131,135,136,137,138,139]. One study showed that 8.5% of clinical isolates contain a duplicated chromosome (commonly 1, 9, 12 or 14), but only 4% of these aneuploid isolates displayed azole resistance [139]. Partial and full chromosomal duplications in clinical populations reduce fitness in vivo [139]. In total, 43.75% (7/16) of azole-resistant *A. fumigatus* chronic pulmonary aspergillosis clinical isolates, which do not possess a mutation in *cyp51A*, display aneuploidy of chromosomal regions containing genes associated with azole resistance, *cyp51A*, *cyp51B* or *cyp51ec*, as well as those encoding MFS and ABC transporters and transcription factors [120].

In *C. albicans* clinical isolates, azole resistance can arise from large genome rearrangements, including the translocations of chromosomal arms; the duplication of the chromosomal region of the left arm of chr5 containing *ERG11* and *TAC1* to produce an isochromosome (i(5L)); trisomies of chr3, chr4, chr5 or chr7; and loss-of-heterozygosity in the chromosomal regions containing *TAC1* (chr5) and *MRR1* (chr3); and the formation of new chromosomes via the duplication of segments containing a centromere and the addition of telomeric ends [27,75,121,126,140,141,142,143,144,145,146,147,148]. Long repeat sequences drive the plasticity of the *C. albicans* genome; for example, the recombination of a long-inverted repeat sequence at the centromere of chr5 is required for the formation of i(5L) [146,149]. Resistance can be attributed to the increased gene dosage of *CDR1*, *CDR2*, *CRZ1* (transcription factor) and *MRR1* on chr3 and *TAC1* or *ERG11* on chr5 [146]. One study predicted that at least 50% of fluconazole-resistant isolates are aneuploid [140]. Recently, a study by [150] showed that different concentrations of fluconazole can select for different genotypic outcomes. Lineages of *C. albicans* that evolved in fluconazole concentrations close to the MIC_50_ of their ancestor acquired aneuploidies and copy number variations, whereas lineages evolved above the ancestral MIC_50_ acquired mutational changes [150]. In *C. albicans*, resistance to posaconazole generated through in vitro experimental evolution also results in aneuploidy and cross-tolerance to fluconazole [145]. Loss of chr5 or combined trisomy of the right arm and monosomy of the left arm of chr5 also leads to caspofungin resistance in *C. albicans* [151,152].

Azole-resistant strains selected directly in vitro or during microevolution experiments also gain permanent aneuploidies [153]. *A. flavus* strains, selected for voriconazole resistance, contain duplications of chromosome 8 or a segmental duplication of chromosome 3, which contains *atrA* but no *cyp51A* mutations [154]. Numerous recent studies on *C. auris* that sequenced and compared the genomes of parental fluconazole-susceptible strains and experimentally evolved fluconazole-resistant strains showed the rapid generation of the aneuploidies of chromosome 5 (which contains the regulator *TAC1B*) or 3, segmental aneuploidy of chromosome 1 (contains *ERG11*), loss of subtelomeric regions, karyotype alterations and the generation of supernumerary chromosomes (centromere-inclusive chromosomal duplications of segments of chromosome 5) [77,108,123,155]. Aneuploids are also commonly found in microevoution experiments on fluconazole resistance in *C. glabrata* [30]. Fluconazole-resistant *C. neoformans MSH2* strains possess permanent aneuploidies of chromosomes 1 and 4 [116].

## 10. Conclusions

There is a critical need for the development of novel antifungals given the restricted number available, limitations on use and the emergence of resistance. Resistance is rapidly becoming an important issue that will worsen without the introduction of new antifungals for use in the clinic. Mutations in key resistance genes such as *ERG11* were once thought to be the predominant cause of antifungal drug resistance. However, the recent use of whole genome sequencing has shown that the microevolution of resistance is far more complicated, and there is still a long way to go to understand this process. Although mutations in single genes such as *ERG11* and *PDR1* are the predominant cause of resistance in *Candida* species, a large proportion of clinical isolates of other fungal species lack *ERG11*-dependent resistance mechanisms and instead possess accumulated mutations in many genes in order to generate a polygenic resistance phenotype. Currently, it is impossible to determine the precise contribution, if any, of every mutational change observed in the genomes of resistant strains. Sexual outcrossing is not possible for most pathogenic fungi, meaning that the association between mutations and resistance phenotypes is difficult to analyse. Their phenotypic contribution to resistance could be confirmed by regenerating the mutation in an antifungal-susceptible strain using gene editing technology; however, this process would be unrealistic to perform for such a large number of mutations. In addition, these complex mutational profiles are coupled with highly plastic genomes—where aneuploidy is rapidly generated either transiently or permanently—and transcriptional changes, which must be separated from adaptive responses. Understanding the many factors contributing to the emergence of resistance is crucial for the development of effective future treatment strategies.

## Data Availability

Not applicable. All articles included in the review are openly available in the NIH National Library of Medicine (https://pubmed.ncbi.nlm.nih.gov/).
